# Birdshot chorioretinopathy: current knowledge and new concepts in pathophysiology, diagnosis, monitoring and treatment

**DOI:** 10.1186/s13023-016-0429-8

**Published:** 2016-05-12

**Authors:** Evangelos Minos, Robert J. Barry, Sue Southworth, Annie Folkard, Philip I. Murray, Jay S. Duker, Pearse A. Keane, Alastair K. Denniston

**Affiliations:** Department of Ophthalmology, Queen Elizabeth Hospital Birmingham, University Hospitals Birmingham NHS Foundation Trust, Birmingham, UK; Academic Unit of Ophthalmology, Centre for Translational Inflammation Research, University of Birmingham, Birmingham, West Midlands B15 2WB UK; Birmingham & Midland Eye Centre, Sandwell & West Birmingham Hospitals NHS Trust, Birmingham, UK; Birdshot Uveitis Society, London, UK; New England Eye Center, Tufts Medical Center, Tufts University School of Medicine, Boston, USA; NIHR Biomedical Research Centre for Ophthalmology, Moorfields Eye Hospital NHS Foundation Trust and UCL Institute of Ophthalmology, London, United Kingdom

**Keywords:** Birdshot chorioretinopathy, HLA-A29, Immunomodulatory therapy, Multimodality diagnostic imaging, T-helper 17

## Abstract

Birdshot chorioretinopathy (BCR) is a rare form of chronic, bilateral, posterior uveitis with a distinctive clinical phenotype, and a strong association with HLA-A29. It predominantly affects people in middle age. Given its rarity, patients often encounter delays in diagnosis leading to delays in adequate treatment, and thus risking significant visual loss. Recent advances have helped increase our understanding of the underlying autoimmune mechanisms involved in disease pathogenesis, and new diagnostic approaches such as multimodality imaging have improved our ability to both diagnose and monitor disease activity. Whilst traditional immunosuppressants may be effective in BCR, increased understanding of immune pathways is enabling development of newer treatment modalities, offering the potential for targeted modulation of immune mediators. In this review, we will discuss current understanding of BCR and explore recent developments in diagnosis, monitoring and treatment of this disease.

*Synonyms for BCR*: Birdshot chorioretinopathy, Birdshot retinochoroiditis, Birdshot retino-choroidopathy, Vitiliginous choroiditis.

*Orphanet number*: ORPHA179

*OMIM*: 605808.

## Background

Birdshot chorioretinopathy (BCR) is a bilateral, autoimmune posterior uveitis with a distinct clinical phenotype and a strong association with HLA-A29. In the early stages of disease, patients often report only mild symptoms, and there may be a significant delay in diagnosis. Unfortunately, the disease is chronic, often progressive and has significant potential for irreversible tissue damage and visual loss.

Historically, the first recognition of BCR as a distinct entity was probably the description by Franceschetti and Babel in 1949 of “candle wax spot chorioretinopathy” in which they reported a 65-year-old woman with discrete depigmented lesions [1]. The first use of the term “birdshot retinochoroidopathy” was in 1980 when Ryan and Maumenee described 13 patients with a distinct syndrome characterized by a white, painless eye with minimal anterior segment inflammation, but with vitritis, retinal vascular leakage and cream-coloured spots at the level of the retinal pigment epithelium (RPE) or deeper layers [2]. In 1981 Gass commented that this phenotype should be extended to include those patients with a similar phenotype but in whom the spots were larger, and where significant depigmentation occurred. In his series of 11 patients, he proposed the name ‘vitiliginous chorioretinitis’ due to the similarity to areas of cutaneous depigmentation seen in vitiligo of the skin [3]. In 1982 Oosterhuis, Baarsma, and Polak used the term “Birdshot chorioretinopathy-vitiliginous chorioretinitis” to describe the syndrome in a further case-series of 6 patients [4]. Since this recognition of BCR as a distinct syndrome in the early 1980s, there has been major progress in a number of areas, notably the recognition of an immunogenetic contribution to its pathogenesis via HLA-A29 and the discovery of novel techniques for phenotyping and monitoring the disease. Although treatment in BCR remains difficult, and often controversial, this continued progress in the recognition and understanding of the clinical phenotype and insights into its pathogenesis provide hope for more effective targeted treatments for patients with BCR in the future.

### Epidemiology & Demographics

BCR is a rare cause of uveitis, although achieving precise estimates of incidence and prevalence data is difficult. Studies from Europe and the USA report that BCR forms between 0.5 and 1.5 % of the uveitis cases seen in specialist uveitis practices [5–9]. Prevalence estimates for uveitis vary considerably according to the population surveyed, but most estimates for Europe and the USA fall between the 38/100,000 reported by Vadot et al. [10] and the 115/100,000 reported by Gritz and Wong [11]. This would suggest that the population prevalence would fall within the range 0.2–1.7 cases/100 000. The actual population prevalence is likely to be at the lower end of this range since most reports on BCR are based on surveys in tertiary centres. Such studies tend to over-represent posterior uveitis syndromes such as BCR. McCannel et al. showed that whilst posterior uveitis formed 14.6 % of 213 consecutive cases of uveitis seen in a university setting, posterior uveitis formed only 4.7 % of uveitis cases seen by community-based ophthalmologists [12]. This would suggest that the population prevalence for BCR is likely to be in the range 0.1–0.6 cases/100 000. Interestingly this is also supported by the population based study by Gritz and Wong of 731 898 people in Northern California, which recorded only one case of BCR in the whole population, equating to 0.14 cases/100 000 [95 % CI 0.0035–0.76] (personal communication reported in Shah et al. [13] supplementary to the study report). In summary the actual prevalence of BCR is uncertain but is likely to be less than 1 per 100 000, possibly in the range 0.1–0.6/100 000. By way of comparison it should be noted that the leading retinal disease age-related macular degeneration has a global prevalence of around 8690/100 000 in those aged 45 or over [14].

BCR is predominantly seen in the middle-aged and in some reports appears to be more common in females. In the landmark systematic review in 2005, Shah et al. reported mean age of disease onset of 53.0 years (512 patients), and a 54.1 % female preponderance (522 patients) [13]. More recently Faia revisited this, supplementing the original data from Shah et al. with subsequent and contemporaneous studies not included in that paper, theoretically extending the series to over 1100 patients (1157 for gender and 1147 for age) [15]. Caution is needed however as a number of these series are from the same centres and it is highly likely that there is some overlap between them. This extended series does however lead to similar estimates with a mean age of onset of 53.3 years and a 58 % female preponderance. Although studies consistently report a mean age of onset in excess of 45 years of age [15], there are occasional reports of younger patients including one of 15 years of age [16] and one of 6 years of age [13].

BCR is most prevalent in Caucasian populations, being most commonly diagnosed in people of Northern European ancestry, with only occasional case reports of BCR in Latino-Hispanic, African-American and Japanese people [17–19]; there is only one report in south Asian populations and this would appear to be an outlier [20].

This ethnic distribution is also relevant to an analysis of the association with HLA-A29 subtypes. At least 17 subtypes have been described, with HLA-A*29.02 and HLA-A*29.01 being the most common in the healthy population positive for HLA-A29 [21]. The HLA-A*29.02 subtype is strongly associated with BCR, being observed in over 95 % of patients [6–9],. whereas the HLA*29.01 subtype is rarely associated with the condition [8–10].. In a study of an ethnically diverse population in the USA, the gene frequency in Caucasians was 4.3 % HLA-A*29.02 vs 0.2 % HLA-A*29.01; in Asians the overall frequency of HLA-A29 was lower, but with HLA-A*29.01 predominating (1.3 % HLA-A*29.01 vs 0.4 % HLA-A*29.02 [22].. This led to the suggestion that HLA-A*29.01 might be protective, and might explain the differences in prevalence between ethnicities. This would not however explain the rarity of the condition among African-Americans and Hispanics in whom the HLA-A*29.02 allele is the most prevalent (3.6 and 4.9 % for HLA-A*29.02 respectively); also, although rare, HLA-A*29.01 has been occasionally observed in Caucasian patients with the disease, being identical to the HLA-A*29.01 haplotype in healthy Asian patients [23]; finally it should be noted that HLA-A*29:01 and HLA-A*29.02, differ by only one amino acid, and this does not appear to affect peptide binding. An HLA-A*29:10 haplotype has also been reported as being occasionally seen in BCR patients [24]. It is clear that HLA-A29 alone cannot fully explain susceptibility to BCR.

### Pathophysiology

Whilst the association of BCR and the haplotype HLA-A*29.02 is well recognised, the exact role of the HLA-A29 molecule in BCR pathogenesis remains poorly understood, and the nature of the other modifying factors that either augment or protect against the effect of HLA-A29 has been unclear. Significant progress has however been made in recent years, which may unpick the chain of events that lead from a class I MHC haplotype to a sight-threatening immune response in the eye [25].

The very strong association of HLA-A29 with BCR was first described by Nussenblatt in 1982 [26]. Although it has been suggested in the past that the apparent association with HLA-A29 was in fact due to linkage disequilibrium with the actual causative gene(s) [27], recent studies have continued to affirm that this is a true association, and that the HLA-A29 gene itself is central to the pathogenesis of the disease [23, 24, 28]. Furthermore Szpak et al. reported on an HLA-A29 transgenic mouse developed using cDNA from a patient with BCR which spontaneously developed a mild chronic posterior uveitis with some similarities to BCR [29]. More recently concern has been raised as to whether this was truly an inflammatory manifestation of the HLA-A29 itself, or whether this was a degenerative process due to the common *Rd8* mutation of the Crb1 gene of C57BL/6. Mattapallil et al. noted that the original strain by Szpak et al. had been lost, but that the *Rd8* was present in most substrains [29, 30].

The role of HLA-A29 was however firmly underlined by a Genome Wide Association Study (GWAS) of Northern European patients and controls, which is noteworthy for two reasons: first it observed an association with HLA-A29 with a p value of 7 × 10^−74^ for HLA-A*29.02; and second it identified a new susceptibility locus, Endoplasmic Reticulum Aminopeptidase 2 (ERAP2). Extending their original GWAS, Kuiper et al. confirmed the association with ERAP2 in a UK cohort, with a combined p value of 2 × 10^−9^ [28].

This association is intriguing as ERAP2, along with the similar ERAP1, is a key enzyme in the processing of antigen to generate suitable peptides for presentation by class I MHC molecules [31]. There are important differences between ERAP1 and ERAP2, such that some antigens can only be processed by ERAP2, as reviewed by Kuiper et al. [28]. The interaction of ERAP1, ERAP2 or both, has now been recognized in a number of other conditions associated with class I MHC such as ankylosing spondylitis, Crohn’s disease and psoriasis. There is thus strong evidence that selective antigen processing by ERAP2, combined with the unique binding motif of HLA-A29 enables a distinct immunogenic signal that lies at the heart of the pathogenesis of BCR.

The missing ingredient in this model is the antigen. Class I MHC molecules have an important role in presenting viral antigens to CD8+ T cells [32]. HLA-B27 has been shown to have a key role in eliminating specific viruses (which may also explain why it is retained in the population), and it is proposed that HLA-A29 may be similarly effective. Kuiper et al. suggest that, due to hypothesized similarities between viral antigens and normal ocular antigens, this powerful anti-viral response may lead to the collateral generation of anti-self CD8+ T cells, and that this triggers the subsequent immune response manifest as BCR [25]. This is an attractive hypothesis, and although neither the putative viral trigger nor the ocular antigen have been identified, it is possible to use new insights from the nature of the HLA-A29 molecule and the ERAP2 molecule to screen for candidates. This has recently been reviewed by Kuiper et al. who note the following: over 100 endogenous ligands for HLA-A*29:02 have been identified, exhibiting considerable variation in residues but all containing tyrosine at anchoring position 9 (P9); the presence of a tyrosine at P9 allows viral and tumour-derived peptides to be recognized by cytotoxic T cells when presented by HLA-A29; such viral antigens include latent membrane proteins (LMP 1 and 2) from Epstein Barr Virus (EBV), several HIV derived proteins and the Vaccinia virus C12L protein; potential ocular antigens include the retinal specific S-antigen and a number of melanocyte derived peptides [25].

One of the challenges of identifying the ‘causative’ ocular antigen is that once inflammation has started there is likely to be exposure of multiple highly immunogenic antigens such as retinal S-antigen and Intraretinal-Binding Protein (IRBP), resulting in extensive retinal autoimmunity and ultimately extensive tissue damage to the eye. Sequences from retinal S-antigen have been shown to bind efficiently to HLA-A29, and in vitro responsiveness to retinal soluble antigen can be demonstrated in a high proportion of BCR patients [26, 33, 34]. It should be noted that peptide fragments will also be presented in the context of other HLA antigens, including HLA class II on antigen presenting cells (APC) [25].

With regard to the possible role of retinal S-antigen, Kuiper et al. point out that, although S-antigen is well-known to be uveitogenic in animal models and responsiveness to S antigen may be observed in many patients with uveitis (not only BCR), this may be a downstream phenomenon arising as a consequence of retinal damage [25]. They particularly draw attention to the possible role of melanocyte derived antigens noting reports of association with vitiligo [3, 35] and other skin diseases and that there appears to be a higher than expected rate of skin (and other) tumours in patients with BCR [36].

A possible additional modifier proposed by Levinson et al., is the presence of selected Killer Immunoglobulin-like receptors (KIRs) on the immune cells of patients with BCR [37]. KIRs are inhibitory and activating receptors expressed on human natural killer (NK) cells and some CD4+ and CD8+ T-lymphocytes, including CD8+ T-lymphocytes, which are important in both innate and adaptive immunity. These allelic combinations are thought to be responsible for altered immune regulation by T-lymphocytes, which is thought to contribute to development of disease. Similarly, other KIR gene alleles appear relatively protective [37, 38]. Levinson et al. reported on the stimulatory KIR haplotype combinations and interaction with HLA-B44 in BCR patients, possibly resulting in loss of self-tolerance during inflammatory conditions and, thus, suggested a role for HLA-B44 in BCR, beyond the strong linkage disequilibrium with HLA-A29 [39]. Further research is required to better understand the underlying mechanisms of HLA class I interacting molecules and elucidate their contribution to BCR pathology [40].

Once initiation of the aberrant immune response of BCR occurs, the door is opened to many of the inflammatory and immune sequelae seen in other forms of autoimmune disease including both animal models of uveitis and human disease. Evidence for T cells being a major player in BCR come from tissue specimens in which they are the dominant cell in the Birdshot lesions [41, 42] and from vitreous fluid samples in which CD4+ and CD8+ T cells predominate [43].

Of particular relevance to BCR are those T cell responses characterized by the secretion of IL-17; Kuiper et al. note that in addition to the well-described Th17 pathway there may be a role for the more recently recognized IL-17-secreting CD8 T cells, ‘Tc17’ cells. IL-17 is significantly elevated in the aqueous humour of patients with BCR [44], and the cytokines associated with the differentiation of naïve T cells to Th17 (IL −23, IL1beta, IL-6 and Transforming Growth Factor-beta (TGFb) have been shown to be elevated in serum and ocular fluids from patients with BCR [44, 45]. Furthermore when peripheral blood mononuclear cells (PBMC) from patients with BCR are stimulated in vitro by retinal antigens, an elevation of Th17 cells with accompanying IL-17 secretion is observed [46].

Tc17 cells have been reported to be critical in the induction of Th17 responses in the animal model experimental autoimmune encephalomyelitis (EAE) [47]. They have been found to be elevated in the blood of patients with BCR [48], and would potentially be a direct connection between the aberrant HLA-A29/ERAP/antigen interaction and a class I restricted pathogenic T cell-mediated response [48].

Other forms of T cell response that may be relevant in the pathogenesis of BCR are the regulatory T cell (T reg) pathways. Although the role of T regs has been considered extensively in uveitis, there is little data looking specifically in BCR other than the report by Foster et al. noting a lower percentage of CD4+ CD25 + FoxP3+ T regs in five patients with BCR compared to controls [49, 50]. Since T reg function declines with age, it is possible that this loss of regulation explains the relatively late onset of the condition in genetically predisposed individuals.

There is continuing debate as to whether BCR is primarily a disease of the choroid or the retina. The indistinct appearance of the lesions, lack of associated RPE pigmentary changes, and the angiographic features of the lesions, suggest these lesions are located in the deep choroidal stroma and are associated with the choroidal veins. Furthermore in the two case reports of enucleated eyes from HLA-A29+ patients, the dominant finding was of focal non-granulomatous T cell infiltrates scattered throughout the choroid. Keane et al. examined the choroid in vivo using extramacular enhanced depth OCT (EMEDOCT), and reported hyperreflective foci which they proposed were likely to be lymphocytic aggregates, with choroidal lesions being noted generally to be located near larger choroidal vessels [51].

Intriguingly the retinal findings, which may be extensive, do not co-localise with the choroidal changes [51]. This may be seen when comparing the atrophic spots seen on fundus autofluorescence with the creamy birdshot lesions seen clinically and recorded on fundus photography; OCT studies highlight these differences at the ultrastructural level and are discussed in more detail later. It is important however to recognize that the retinal and choroidal changes are not necessarily concordant, and that this reflects both our ability to monitor the disease and may indeed reflect different aspects of its pathogenesis.

### Clinical presentation

#### Clinical symptoms

The disease is bilateral and commonly symmetric, although asymmetric involvement is sometimes noted. Early in its presentation, patients may report a range of visual symptoms, the seriousness of which may not be recognized, particularly as visual acuity (VA) is often preserved [13, 17, 36, 52–55]. In the review by Shah et al., they note that of 126 patients for whom data related to symptoms was available, 88 % reported blurred vision, 43 % floaters, 18 % nyctalopia and 9 % dyschromatopsia [13]. Importantly, of the 13 patients with 6/6 vision or better in both eyes, 12 (92 %) had visual complaints at presentation with 10 (83 %) reporting blurred vision despite the apparently good acuity. It is likely that this represents an awareness of loss of contrast sensitivity, presence of metamorphopsia or presence of small scotomata. Additional features noted in this and other series include glare, photopsia, photophobia, reduced peripheral vision, metamorphopsia and decreased depth perception [13].

In their series, Rothova et al. noted that subjectively ‘blurred’ vision is present in 68 % (despite preservation of good VA), floaters in 29 %, nyctalopia in 25 %, reduced contrast sensitivity in 20 %, dyschromatopsia in 20 %, glare in 19 %, reduction in peripheral vision in 19 % and photopsia in 17 % [36].

#### Clinical signs

**Fig. 1:**
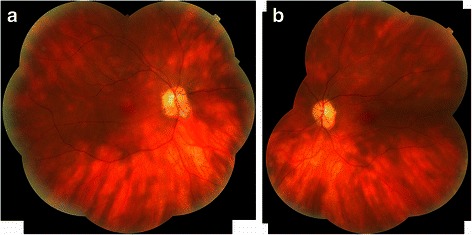
Fundus photomontage of right (**a**) and left (**b**) eyes of a patient with BCR revealing both classic creamy ovoid lesions and the linear streaks of more advanced lesions

**Fig. 2:**
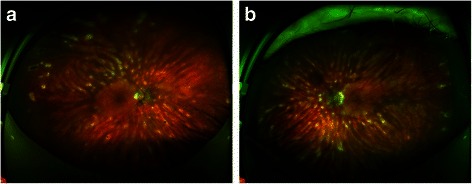
Wide-field imaging such as with the Optos™ of right (**a**) and left (**b**) eyes of a patient with BCR is helpful in revealing the distribution of lesions, and may make the diagnosis more obvious than on standard field fundus photography

Anterior segment signs are generally absent, although a mild anterior uveitis is sometimes observed. A mild vitritis, without demonstrable snow banking or snowballs, is reported in up to 83 % of cases according to Priem and Oosterhuis [53]. Fundoscopy classically reveals characteristic creamy ovoid choroidal lesions, measuring 500–1,500 μm in diameter (Figs. 1 and 2). These lesions give the typical ‘Birdshot’ appearance responsible for the name of the condition, but may not be apparent in the early stages of disease, with a lag time of up to 8 years reported after initiation of symptoms according to Godel et al. [54] As BCR progresses these lesions become more confluent, coalescent and form linear patterns around retinal veins. As they become more advanced, they become more atrophic in appearance (Figs. 1 and 2). Extensive posterior lesions can give the appearance of peri-papillary atrophy suggesting the presence of other causes of multi-focal choroiditis like histoplasmosis.

Development of CMO is common and is the leading cause of visual loss in patients with BCR. In the series of 102 patients reported by Priem and Oosterhuis, CMO occurred in 63 % of cases, retinal vasculitis in 40 %, disc oedema in 38 %, cellophane maculopathy in 10 %, retinal neovascularization in 7.5 %, choroidal (‘sub-retinal’) neovascularization and macular scar in 6 % and optic atrophy in 4 % [53]. In a series of 37 patients with five years of follow-up since diagnosis of BCR, Rothova reported that the major complications of BCR were CMO (84 %), cataract (60 %), glaucoma (19 %) and choroidal/retinal neovascularization (14 %) [36].

### Diagnosis

Over the years a number of diagnostic criteria have been proposed. The original description by Ryan and Maumenee listed: (1) White, painless eye; (2) minimal, if any, anterior segment inflammation; (3) diffuse vitritis without snowballs or snowbanking; (4) retinal vascular leakage, particularly in the posterior pole, which may be associated with macular oedema and optic disc oedema; (5) distinctive, discrete, cream coloured or depigmented spots throughout the fundus [2]. Later, Priem and Oosterhuis suggested an abridged version of these criteria based on their observation of 102 cases of BCR. They suggested minimal criteria as being: (1) Bilateral typical birdshot lesions with (2) two or more of (i) vitritis, (ii) cystoid macular oedema, (iii) arteriolar narrowing and irregularity of the veins, (iv) retinal vasculitis, (v) disc oedema, (vi) cellophane-like maculopathy, (vii) retinal neovascularization, (viii) choroidal (‘subretinal’) neovascularization and macular scar, and (ix) optic atrophy [53].

These early diagnostic criteria have since been superseded by the recommendations of the International workshop held at UCLA [55]. Essential criteria are (1) bilateral disease (2) three or more characteristic birdshot lesions inferior or nasal to the disk in one eye, (3) low-grade anterior chamber inflammation (no more than 1+ cells in the anterior chamber on the SUN score), (4) low-grade vitreous inflammation (no more than 2+ on the NEI/SUN vitreous haze score). Birdshot lesions were defined as being “cream-coloured, irregular or elongated, choroidal lesions with indistinct borders, the long axis of which is radial to the optic disk”. Additional supportive findings include the presence of HLA-A29 positivity, retinal vasculitis, and CMO. Exclusion criteria include keratic precipitates, posterior synechiae and the presence of infection, neoplastic disease, or other inflammatory causes of multifocal choroidal lesions (Table 1).

**Table 1:** Summary of research diagnostic criteria for BCR as defined at the 2006 UCLA international workshop [28]

Required characteristics	Disease in both eyes
	≥3 peripapillary birdshot lesions (cream-colored, irregular or elongated choroidal lesions with long axis radiating from optic disc)
	≤1+ anterior vitreous cells
	≤2+ vitreous haze
Supportive characteristics	HLA-A29+
	Retinal vasculitis
	Cystoid Macular Oedema (CMO)
Exclusion criteria	Keratic precipitates
	Posterior synechiae
	Presence of infectious, neoplastic or other inflammatory diseases that can cause multifocal choroidal lesions

#### Differential diagnosis

The differential diagnosis for BCR is shown in Table 2. In most cases the clinical pattern of BCR is distinct, and can be easily differentiated from other ‘white dot’ syndromes. The leading differential diagnosis of a ‘typical’ BCR presentation is sarcoidosis [56], although tuberculosis, syphilis and ocular lymphoma [57] should also be considered, particularly in those cases where the chorioretinal lesions are less typical of the small ovoid ‘Birdshot’ lesions.

**Table 2:** Differential diagnosis of Birdshot Chorioretinopathy

Infectious	Tuberculosis
	Syphilis
	Ocular Histoplasmosis Syndrome
Non-infectious	Sarcoidosis^*^
	Vogt-Koyanagi-Harada syndrome (VKH)
	Sympathetic Ophthalmia
	Acute Posterior Multifocal Placoid Pigment Epitheliopathy (APMPPE)
	Multiple Evanescent White Dot Syndrome (MEWDS)
	Multifocal Choroiditis and Panuveitis Syndrome (MCP)
	Punctate Inner Choroidopathy (PIC)
Masquerade	Lymphoma^*^

These conditions may mimic some aspects of BCR, although few of these will cause diagnostic confusion. The conditions which can most closely resemble BCR are marked with an asterisk (^*^)

As with all cases of posterior segment involving uveitis, we recommend a careful clinical assessment (history, examination and investigations as needed) to exclude infection and systemic disease. Although there is no ‘diagnostic’ test of BCR, laboratory investigation and multimodal imaging may be supportive. In patients presenting with a typical clinical appearance of BCR, we would routinely undertake the following tests: HLA-A29, syphilis serology, ACE level in all cases; interferon gamma release assay and/or a Mantoux test in selected high-risk cases; chest X-ray (looking for evidence of sarcoidosis or TB) in all cases; Indocyanine green angiography (ICG), fluorescein angiography (FA), and electroretinography (ERG) in all cases. Although the ICG, FA and ERG findings in BCR are not unique, they may support the diagnosis and have value in monitoring the disease and assessing response to therapy (discussed later) and so are useful as a baseline assessment. Additionally we would undertake baseline haematological and biochemical analysis with a view to the likelihood of undertaking systemic immunosuppression; specifically we would perform full blood count, urea and electrolytes, liver function tests, glucose, lipids, and haemoglobin A1c (HbA1c).

For some conditions, the passage of time may also help identify the underlying disease. Thus conditions such as sarcoidosis, tuberculosis and syphilis may initially present with a limited posterior uveitis with some resemblance to BCR but then progress to a more extensive ocular and/or systemic phenotype that is clearly incompatible with BCR. For example the development of a significant anterior uveitis with mutton fat keratic precipitates and posterior synechiae would exclude the diagnosis of BCR, but would be compatible with sarcoidosis or tuberculosis [30]. Conversely it should be noted that the late presentation of a patient with advanced BCR may itself lead to diagnostic confusion, such as a case of advanced retinal changes in BCR mimicking retinitis pigmentosa as reported by Willermain et al. [58].

Some white dot syndromes can be distinguished with relative ease on clinical examination. For example, in Acute Posterior Multifocal Placoid Pigment Epitheliopathy (APMPPE), the usual fundal lesions are described as displaying placoid morphology, and are located predominantly in the posterior pole. On clinical investigation, fundal lesions in APMPPE tend to exhibit characteristic angiographic features of early blockage and late staining (“block early and stain late”). Moreover, the acute lesions of APMPPE typically show clinical resolution, leaving areas of retinal pigment epithelial hyperpigmentation, whereas the retinal lesions BCR do not [59, 60]. Other important white dot syndromes to distinguish from BCR include Multifocal Choroiditis with Panuveitis (MCP), which exhibits smaller, discrete, punched out hyper- and hypopimented lesions which typically show early blockage and late staining on fluorescein angiography, and are predominantly located around the optic disc [61].

Choroidal lesions appearing in the uveitic phase of Vogt-Koyanagi-Harada (VKH) disease may be distinguished from those of BCR by the presence of associated exudative retinal detachment. Furthermore, lesions of VKH display characteristic pinpoint areas of hyperfluorescence at the level of the RPE with subneurosensory pooling on fluorescein angiography. In addition, VKH is a systemic disease with characteristic extraocular differentiating features [62].

##### Diagnostic value of HLA-A29

HLA-A29 testing must be used with an understanding of where it is of greatest diagnostic value. It has been shown that whilst HLA-A29 is detected in almost 96 % of patients with BCR, the positive predictive value of HLA-A29 as a screening test in patients with posterior uveitis remains less than 50 % due to the rarity of BCR (rare even within the posterior uveitis population) and the background prevalence of HLA-A29 in the rest of the population (eg up to 5 % for Caucasians as discussed earlier). Routine screening of HLA-A29 status in uveitic patients is therefore discouraged [13, 35, 36]. In our opinion HLA-A29 screening is however useful for patients with bilateral multifocal choroiditis and clinical features consistent with a diagnosis of BCR. Although HLA-A29 is not an absolute criterion for the diagnosis of BCR [33, 55], it has been suggested that the negative predictive value of HLA-29 typing in this cohort is as high as 99 % and thus a diagnosis of BCR is highly unlikely in the absence of HLA-A29, and should prompt extensive work-up for other conditions that may mimic BCR as outlined earlier.

### Treatment

The mainstay of treatment in BCR is steroid-sparing immunomodulatory therapy (IMT). Gasch et al. suggest that up to 20 % of disease is self-limiting, with eventual complete remission [17]. Despite this, long-term follow-up suggests that the majority of cases are characterised by multiple inflammatory exacerbations with progressive visual loss resulting from structural complications and global retinal dysfunction [36, 53, 63, 64]. There is not yet consensus regarding the optimal treatment regime or duration of therapy for patients with BCR, with centres managing these cases developing localised algorithms for best therapy according to their experience and the limited published data available.

It is common practice for systemic corticosteroids to be employed as initial or rescue therapy in the management of acute inflammatory manifestations of the disease, but these are best considered a bridging therapy until systemic IMT becomes effective. Indeed, several groups have demonstrated that early and sufficiently dosed immunosuppressive treatment can prevent the appearance of typical BCR fundus lesions [65, 66]. Periocular and/or intravitreal injections are commonly employed first-line for the treatment of acute or recurrent macular oedema [67, 68].

The data on the use of long-term systemic corticosteroid therapy is mixed. Kiss et al. presented a retrospective case series of 28 patients with a mean follow-up of 81.2 months, concluding that systemic corticosteroids are of inconsistent efficacy when used as monotherapy, necessitating unacceptably high maintenance doses associated with development of serious steroid-associated adverse effects [67]. Becker et al. reached similar conclusions in their review [63]. In one series, Thorne et al. reported outcomes of 40 BCR patients, concluding that fewer than 15 % of BCR patients remain in regression with doses of systemic prednisolone monotherapy of less than 20 mg/day [68]. Sporadic case reports are however documented in the literature reporting treatment success with maintenance doses as low as 5 mg/day [69].

Options for steroid-sparing IMT include antimetabolites eg methotrexate (MTX), mycophenolate mofetil (MMF),T-cell transduction/calcineurin inhibitors (eg cyclosporine A (CsA)), intravenous immunoglobulin (IVIg) and other biologic therapies, each of which may be used alone or in combination with other agents.

As discussed previously, evidence of T-cell-mediated pathology in BCR patients has supported the use of CsA in patients for whom low-dose prednisolone is insufficient to control their intraocular inflammation [70, 71]. Although this has proven effective in control of BCR, its use is limited by side-effects including renal impairment and hypertension which tend to cause more significant problems in the predominantly middle-aged population of BCR patients [36, 61, 64]. These effects may be reduced with low-dose therapy. Vitale et al. reported a reduced rate of side-effects in a case series of 8 patients treated with low dose cyclosporine A monotherapy at doses between 2.5 and 5 mg/kg/day, observing a 25 % incidence of hypertension and no cases of nephrotoxicity [70]. In the retrospective series reported by Kiss et al., 26 of 28 patients receiving IMT for BCR received CsA alone or in combination with MTX, azathioprine, MMF or daclizumab, with favourable visual outcome, inflammatory control, stabilization of ERG parameters, and the absence of demonstrable nephrotoxic side effects [67].

Antimetabolite agents such as azathioprine, MTX, and MMF have been widely used as steroid-sparing agents in the treatment of BCR with varying degrees of success. MMF has become increasingly popular in recent years and has proven effective in the treatment of non-infectious uveitis [72]. Although gastrointestinal side effects are common MMF is generally well tolerated at doses between 1–3 g/day. In their retrospective series, Doycheva et al. examined the long-term efficacy and tolerability of 24 patients with BCR receiving mycophenolic acid derivatives (either MMF or mycophenolate sodium, MPS). They noted that control of intraocular inflammation (defined as absence of clinical and angiographic signs of inflammation) was achieved in 16 of 24 patients (67 %), and with successful corticosteroid tapering to ≤10 mg daily dose in the 20 out of 21 patients who received systemic corticosteroids. Drug-related side effects occurred in 12 patients (50 %, rate 0.16/patient-year), with four patients being switched from MMF to MPS due to gastrointestinal discomfort. [73] The use of MMF in BCR was also supported by Tomkins-Netzer et al. who noted in their retrospective study of 46 patients with BCR, that 86 % of their patients received MMF. [74] A combination regimen of MMF with CsA is also reported to achieve long-term control of inflammation [75]. Although now less commonly used than MMF, MTX also appears to be efficacious in BCR with Rothova et al. reporting better visual outcomes compared to those achieved with either no systemic treatment or corticocosteroids alone [76].

Evidence supporting the use of biologic agents in BCR is limited. Sobrin et al. reported on the use of the anti-IL-2 receptor blocking agent, daclizumab (1 mg/kg every 2 weeks) in the treatment of a small case series of patients with BCR refractory to traditional IMT, with, 7 of 8 patients achieving stabilization or improvement in visual acuity in both eyes with complete resolution of vitreous inflammation, while six achieved fluorescein angiographic resolution of retinal vasculitis and CMO [77]. There was however a decline in 30 Hz implicit times and bright scotopic amplitudes on ERG in some patients, thought to be due to the delay in achieving control of disease. The authors conclude that early and aggressive treatment remains important in BCR. Yeh et al., reported on the use of daclizumab in 2 patients with BCR, achieving more rapid control of inflammation with the use of higher doses of daclizumab (8 mg/kg followed by 4 mg/kg) [78].

The use of the anti-TNF agent, infliximab, in the treatment of refractory BCR cases unresponsive to other immunosuppressants has also been reported. In their series, Artornsombudh et al. reported on 22 patients treated with infliximab, of whom 6 patients discontinued therapy due to development of side effects [79]. Observed side effects included neuropathy, drug-induced lupus, allergic reactions, and secondary fungal infection [79].

A more recent arrival is tocilizumab, a humanised antibody that binds both to soluble and membrane bound IL-6 receptors. It has previously been used in the management of refractory non-infectious uveitis and refractory macular oedema, with some limited experience in BCR refractory macular oedema [80, 81]. Mesquida et al. noted that in 6 eyes of 3 patients with refractory macular oedema due to BCR, control of inflammation and resolution of macular oedema was achieved with tocilizumab in all 6 eyes [80].

As described earlier, there is evidence that the cytokine IL-17 may have a pivotal role in the pathology of uveitis, and BCR in particular. Secukinumab (Novartis International AG) is a high affinity fully human monoclonal antibody that binds and neutralizes IL-17A. After encouraging preclinical and early phase data, three major randomized controlled trials of subcutaneous secukinumab in non-infectious uveitis were conducted (SHIELD, INSURE and ENDURE). The first of these, a study of uveitis associated with Behcet’s disease, failed to achieve its primary efficacy endpoint, leading to the early termination of the other two studies [82]. This appeared to close the door on secukinumab as a treatment for uveitis, but there remained the possibility that this was a bioavailability issue resulting from the use of a subcutaneous preparation rather than the intravenous preparation used in the proof-of-concept study. Indeed in a more recent open label study comparing preparations, secukinumab 30 mg/kg IV and 10 mg/kg IV were associated with higher responder rates than the 300 mg SC dose (72.7 % and 61.5 %; vs. 33.3 %) and higher remission rates (27.3 % and 38.5 %; vs. 16.7 %). Coupled with the evidence that patients with BCR have elevated aqueous IL-17 in BCR and enhanced Th17 responses to retinal antigens in vitro,[[44–46] this more recent study once again opens the door to the possibility that targeting IL-17 remains an important avenue for exploration in the management of BCR.

IVIg has been used with promising results by Cassoux et al. who reported on the outcomes of 66 treated eyes [83]. Efficacy was assessed by measurements of visual acuity and a decrease in inflammation and macular oedema on fluorescein angiograms. They reported stabilization of visual acuity in 19 eyes (29 %) and improvement of visual acuity in 35 eyes (53 %). Macular oedema improved in 65 % according to results on fluorescein angiography with overall control of inflammation in 81 % of treated eyes. Treatment was discontinued in 3 patients due to significant side effects. Reported side effects included transient systemic hypertension, headache, eczematous lesions and hyperthermia [83].

Local therapies are an attractive option in BCR. The fluocinolone acetonide intravitreal implant, Retisert (Bausch & Lomb Incorporated, Bridgewater, NJ, USA), has been used with good results in BCR patients. Burkholder et al. reported their treatment outcomes in a series comprising 20 eyes of 11 patients with BCR, observing resolution of macular oedema in 7/8 patients (88 %) and control of uveitis in all eyes (100 %) [84]. Retisert is associated with a high rate of cataract progression/development and ocular hypertension and secondary glaucoma: in the aforementioned case series, 7 eyes (78 %) required cataract surgery within 3 years and 14 eyes (70 %) required glaucoma surgery. Furthermore, Burkholder et al. observed that post-procedure IOP increases occurred earlier in BCR patients than in comparison groups with other types of autoimmune uveitis, with a median time to development of IOP > 20 mmHg of 5.5 months in BCR patients, compared to 11.5 months in the comparison group [84]. Rush et al. also warn that the optic nerve in BCR patients may also be more vulnerable to damage from a number of factors including decreased optic nerve perfusion arising from reduced choroidal circulation [85].

Iluvien (Alimera Sciences, Alpharetta, GA, USA) is a novel fluocinolone acetonide implant which is licensed for diabetic macular oedema and appears to have a more benign profile than Retisert with reduced rates of elevated intraocular pressure and the major advantage of being injectable via a 25-gauge system. It is currently being evaluated in a Phase III trial for use in posterior segment involving uveitis (pSivida Corp, Watertown, MA, USA [NCT 01694186]) [86].

An alternative corticosteroid implant is the dexamethasone implant, Ozurdex (Allergan, Inc., Irvine, CA, USA). This injectable implant is licensed for use in non-infectious posterior segment uveitis in USA and Europe, an indication which includes BCR. Ozurdex releases dexamethasone in a biphasic manner over 6 months, with higher concentrations released for the first 6 weeks. Although there are no clinical trials specifically evaluating the use of Ozurdex in BCR, there are individual case reports of the use of Ozurdex in BCR [87–89], and it is worth noting that patients with BCR are often significant contributors to studies which support the use of Ozurdex in uveitis. For example in their retrospective cohort studies of Ozurdex in posterior segment uveitis, Zarranz-Ventura et al. reported that 12 of 82 patients enrolled had BCR, and Pelegrin reported 7 of 42 patients with BCR [90, 91].

The major licensing study for Ozurdex in posterior segment uveitis was HURON [NCT00333814], a phase III double-masked, randomized, controlled trial that compared the effect of two implant doses (0.7 mg and 0.35 mg) with sham injection. The HURON reports do not include information on how many (if any) BCR patients were included, and therefore any support for the use of Ozurdex in BCR is indirect. Both implant doses led to reduction in vitreous inflammation, improved visual acuity and reduction in cystoid macular oedema, but with the 0.7 mg implant providing a longer duration of action without a significant increase in side effects; it is this 0.7 mg implant that is licensed as Ozurdex [92].

HURON may however provide valuable data with regard to safety. Ozurdex was associated with increased rates of both cataract and elevated intraocular pressure (IOP), but these increases were modest: at 26 weeks cataract was reported at 15 % in 0.7 mg implant group vs 7 % in the sham group, and IOP of 25 mmHg or greater was reported at 7.1 % in the 0.7 mg implant vs 4.2 % in the sham group [92].

Anti-VEGF therapy such as ranibizumab and bevacizumab appear to be of little value in the management of CMO in BCR; studies of their use in CMO-associated with a range of forms of uveitis suggest that they are well-tolerated but the effect is limited and transient [93–96].

In line with most commentators, we would conclude that local administration of drugs remains an attractive option in BCR, but most commonly as an adjunct to systemic therapy.

In summary the limited data available supports the use of local corticosteroid therapy in BCR particularly in regard to CMO and vitreous haze [84]. Unfortunately other critical indicators of BCR activity and progression (eg visual field sensitivity or 30Hz flicker) are generally not reported in these studies, and therefore it is not yet clear the extent to which local therapy alone may control underlying disease progression.

Overall the need is for more targeted therapies which avoid the commonly experienced side-effects of current therapies. This will only arise through increasing our understanding of the biological processes underlying BCR [97, 98]. Areas of interest include drugs that influence leukocyte migration (fingolimod, natalizumab), target specific cell subtypes (rituximab), alter cell-cell interactions (abatacept), or affect cytokine signaling (gevokizumab, secukinumab). All these and other emerging therapies in uveitis, and BCR in particular, have recently been reviewed [97, 98].

### Prognosis

BCR is a progressive disease with the potential for significant visual impairment due to anatomical and functional complications. Common causes of visual loss in BCR include refractory CMO, macular scarring, development of choroidal neovascular membrane and cellophane maculopathy. Diffuse retinal dysfunction associated with long duration of disease is recognised as a statistically significant risk factor for vision loss.

Many studies have shown that despite the accumulation of irreversible, peripheral retinal damage, central BCVA may remain well preserved until late in the disease course with few patients experiencing permanent visual loss. CMO with associated central visual loss is thought to occur in 10 % per eye-year, and reported incidence rates for the development of vision loss to 20/50 or worse and to 20/200 or worse are 13 and 4 %, respectively [68].

As with many areas in ophthalmology, prospective data with respect to rates of relapse and remission in patients with BCR, as well as to the optimal duration of IMT, is lacking. As previously discussed, fewer than 15 % of patients achieve an adequate clinical result when treated with systemic steroids at maintenance doses of less than 20 mg/day. Furthermore, studies with up to 10 years long-term follow-up of BCR patients show possible progressive retinal dysfunction and poor visual outcomes despite treatment with corticosteroids and/or steroid-sparing IMT. [36, 63]

The definition of ‘disease remission’ can be difficult in a condition such as BCR. Vitale has argued that the Standardization of Uveitis Nomenclature Working Group definition of remission as disease inactivity for 3 months or more following cessation of treatment is not appropriate for BCR. Vitale proposed that it may be more appropriate to use definitions of ‘clinical remission’, as the observation of inactive disease for 6 months on medication, and ‘durable remission’ as the observation of inactive disease off all IMT for 1 year [99].

## Monitoring

Due to the progressive nature of BCR, it is essential to have accurate methods of monitoring disease activity and measuring accumulated damage. Recent technological advances have dramatically increased the range of available modalities we can use both for primary diagnosis of BCR, and for monitoring of disease relapses and remissions. Whilst clinical examination remains the mainstay of diagnosis, this is increasingly supported by laboratory investigation and further supplemented by advanced multi-modal imaging techniques. Multimodal imaging includes FA, ICG, optical coherence tomography (OCT) and fundus autofluorescence (FAF). In addition, ERG and perimetry remain useful adjuncts to diagnosis and monitoring. Despite these advances, monitoring disease activity and progression in BCR remains difficult. The correlation between patient symptoms, clinical findings and ancillary tests may be poor, and all of the ancillary tests have some limitations. In most posterior segment uveitis the common methods of detecting deterioration (at least in clinical trials) are worsening visual acuity, increasing vitreous haze, and presence of CMO [100], but many patients with BCR experience an insidious course in which profound loss of overall visual function may occur despite preservation of central acuity and absence of clinically obvious inflammation. In contrast when disease activity manifests as CMO then the decision to treat may be straight-forward as the patient is likely to be symptomatic and there will be objective evidence on FFA and/or OCT to support it.

### Multimodal Imaging

#### Fluorescein angiography

FA is commonly used in assessment and monitoring of active BCR. Fluorescein angiographic findings of the lesions are inconsistent and depend on the age of the lesions and the phase of the study. Early birdshot lesions demonstrate early hypofluorescence with subtle late staining; this is attributed to inflammatory infiltrate at the level of the outer choroid associated with large choroidal vessels, which is thought to disrupt perfusion of the choriocapillaris, causing a secondary alteration in the RPE. Leakage at the optic nerve is typically seen in late phase images, commonly observed as a segmental periphlebitis. Cystoid macular oedema and choroidal neovascularization may also be evident in later stages of the disease [53, 101–103]. The arteriovenous transit time is frequently prolonged in BCR and it has been suggested that this finding may have diagnostic value; this is attributed to extreme leakage of fluorescein dye from retinal arterioles and diffusion into the surrounding tissue prior to entering the venous circulation [103].

#### Indocyanine green angiography

ICG is more sensitive in revealing multiple hypo-fluorescent spots in the early and middle phase of the study, which are typically distributed around choroidal vessels [104]. Some of these hypo-fluorescent spots correspond to lesions visible on fundoscopy or FA, but otherwise ‘invisible’ lesions also appear to be identified by this method. It is more sensitive than FA in revealing choroidal lesions and is thus thought to be a better measure of disease activity. Some areas of patchy hyperfluorescence observed on FA are noted to correspond to hypofluorescent lesions on ICG [105].

#### Optical coherence tomography

OCT is a non-invasive method used to visualise the retina and choroidal layers, and is widely used in the detection and monitoring of BCR. It is useful in the detection of subtle clinical signs not easily observed on clinical examination; in one study, 31 % of 122 eyes with BCR were found to have macular oedema at baseline using time-domain OCT. [106]

Spectral domain OCT (SD-OCT) is a newer development in OCT imaging which reveals more precisely the inner and outer retinal anatomy, enabling clear identification of the external limiting membrane, the photoreceptor ellipsoid zone, and the RPE/Bruch’s membrane complex. Macular thinning and disruption of the photoreceptor IS/OS junction have been noted using both time domain and SD-OCT, and has been associated with decreased VA, reduced contrast sensitivity and is indicative of a poor visual prognosis [106–108]. Birch et al. reported a strong positive correlation between macular atrophy on SD-OCT, poor VA and depressed multifocal ERG (mfERG) foveal responses in patients with longstanding BCR [107]. SD-OCT enabled better characterisation of macular pathology, illustrating that macular thinning was associated with a loss of thickness of the segment subtending the proximal border of the outer plexiform layer and Bruch’s membrane. SD-OCT findings thus suggest that macular atrophy in BCR occurs largely in the outer retina [107, 108].

**Fig. 3:**
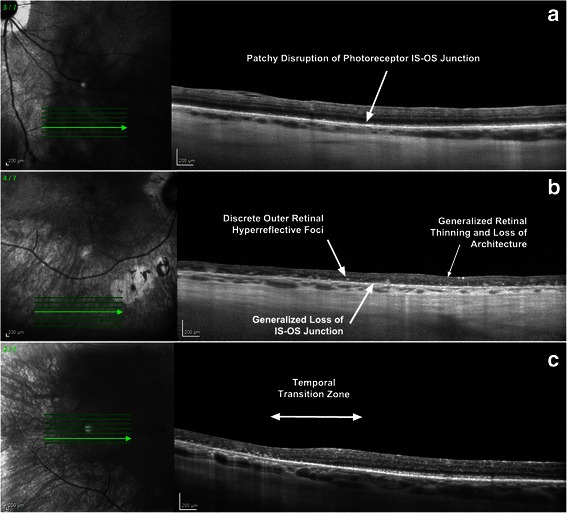
Detection of novel retinal morphologic parameters using extramacular optical coherence tomographic (OCT) scanning protocols. **a** Near-infrared fundus image and inferior extramacular OCT B-scan reveal patchy disruption of the photoreceptor inner segment/outer segment (IS/OS) junction. **b** Near-infrared fundus image and inferior extramacular OCT B-scan reveal generalized thinning/loss of the retinal architecture, generalized loss of the IS/OS junction, and the presence of discrete outer retinal hyperreflective foci. **c** Near-infrared fundus image and temporal extramacular OCT B-scan reveal the transition zone between a grossly normal and a diseased retina. (With permission from Keane et al.) [51]

Recently developed advanced SD-OCT imaging techniques including “enhanced depth imaging” (EDI) protocols [109] and extramacular image acquisition [51, 110] offer high resolution visualisation of the choroidal anatomy and the delineation of potentially significant structural changes outside the macula/vascular arcades respectively, which are not visible by conventional OCT. Keane et al. demonstrated that extramacular image sets revealed a spectrum of outer retinal substructure derangement ranging from focal disruption to generalised loss of the photoreceptor Inner segment/outer segment junction as well as visualisation of a “transition zone” in which structural abnormalities were initially seen (Fig. 3) [51]. In a retrospective study of 14 HLA-A29 positive BCR patients evaluated clinically and with EDI SD-OCT, Birnbaum et al. noted a suprachoroidal fluid band, the presence and thickness of which was positively correlated not only with the subjective complaint of photopsia, but also with overt signs of active inflammation, associated retinal vasculitis and vitritis [111]. This indicates that the use of EDI and extramacular SD-OCT may allow improved phenotyping of posterior uveitic entities including BCR. The utility of EDI in monitoring disease activity will require prospective study to determine the extent to which these choroidal morphological abnormalities may be modified with immune-modulatory therapy [99].

Further advances in OCT - notably Wide-Field SD-OCT, Swept Source-OCT and OCT Angiography - are likely to extend the role of OCT in monitoring disease activity and damage in BCR. De Carlo et al. recently utilized the AngioVue prototype software of the RTVue XR SD-OCT to analyse the retinal and choroidal vasculature in the posterior pole. OCTA enabled in eyes with typical BCR lesions demonstrated areas of decreased choroidal blood flow below the disrupted retinal pigment epithelium; additional features included retinal thinning, telangiectatic vessels, and an increased intercapillary space. Capillary dilatations and loops were each seen in 7 of 8 eyes (88 %). Prospective study is needed to determine the natural history of these changes, their relevance to visual function and their response to treatment [112].

#### Fundus autofluorescence

FAF is an in vivo modality that exploits the autofluorescence properties associated with lipofuscin accumulation within RPE cells and that of other fluorophores within the outer retina and subretinal space. FAF in BCR reveals hypo-fluorescent areas representing RPE atrophy; however, these lesions do not always correlate with lesions visible on fundoscopy. The significance of this discrepancy is unknown.

The presence of linear hypo-autofluorescent streaks on FAF which correspond to visible changes along retinal blood vessels in some patients, is thought to represent retinal vasculitis which is likely to play an independent role in mediating inflammatory damage to the RPE. Additionally, macular RPE atrophy appears to correlate with placoid macular hypo-autofluorescent areas associated with visual acuity equal to 20/50 or worse and with decreased mean foveal thickness as demonstrated on OCT scans [113].

### Electrophysiology

ERG remains the tool of choice for BCR monitoring in many centres. In addition, results of mfERG have been shown to be abnormal even among those patients without evidence of macular atrophy on SD-OCT, suggesting that mfERG changes precede thinning on OCT damage and may serve as a sensitive surrogate marker for disease activity before the development of irreversible structural damage. ERG may therefore also assist in primary diagnosis.

Full field ERG and mfERG are good monitoring tools with good sensitivity, able to detect subtle functional retinal changes and are useful in determining the response to treatment [114–119]. Prolongation of the 30 Hz cone flicker implicit time is a particularly useful marker of disease activity in BCR, and is associated with visual acuity changes or stabilisation [115]. In addition to the 30 Hz flicker implicit time, perturbations in other ERG parameters, such as the decreased dim rod scotopic b wave and decreased bright scotopic b wave amplitudes, have also been shown to correlate with disease severity (night blindness) and treatment failure on tapering of IMT [114, 118]. Full-field ERG in BCR typically demonstrates an initial decreased amplitude and increased latency of the b-wave as well as loss of oscillatory potentials, suggesting dysfunction of the inner retina [114, 115]. As disease progresses, electroretinographic dysfunction also becomes evident in the outer retina. Loss of visual acuity has been observed to lag behind electroretinographic dysfunction.

Longitudinal studies are required to assess the predictive value of mfERG in birdshot patients. A decline in mfERG may precede severe acuity decline and assist the clinician in deciding which patients require additional treatment. In a systematic review by Moschos et al. it is reported that the electronegative ERG pattern associated with selective b-wave amplitude reduction compared to the a-wave amplitude, results in a low b:a ratio which is unique in BCR, and does not appear in any other type of uveitis [117]. Hirose et al. in a study of 15 patients confirm the findings from Moschos et al. which could be a helpful diagnostic ERG sign for BCR detection [118]. These ERG findings indicate that in BCR the neural layers of the retina are more diffusely and severely involved than the receptor-retinal pigment epithelium-choroid complex. In the most advanced stage, the patient becomes nyctalopic with a non-recordable ERG, similar to the situation seen in retinitis pigmentosa [118].

Although a valuable monitoring tool, electrophysiological testing is time- and labour-intensive and currently not available in all centres. Common practice where these tests are available is to perform standard electrophysiological testing including 30Hz flicker on an annual basis, but with additional interim testing where there is concern over possible deterioration in the absence of clinical evidence to direct treatment.

### Perimetry

Perimetry is useful in monitoring peripheral retinal health in patients with BCR; SITA 24–2 is the perimetric method of choice; however Goldmann perimetry may be preferred when the macula is severely affected.

Despite a variation in approach to visual field assessment between different study groups, a consistent observation is that extensive visual field deterioration may occur despite well-preserved central visual acuity [119]. In one study, abnormalities on Humphrey visual field (HVF) testing were present in 62 % of 80 patients with BCR at baseline with the most common patterns being multiple foci and arcuate defects [120]. Whilst mean deviation scores have been shown to correlate with patient symptoms including blurred vision, nyctalopia and poor contrast sensitivity, there is less association with visual acuity. In addition, total deviation has been shown to correlate with disappearance of the inner segment/outer segment band on time domain OCT. In one study by Thorne et al., Goldmann visual fields defects (in I4e isopter) were detected within 6 months of presentation in 75 % of patients [121]. In this study, continued visual field loss was also observed among those receiving no treatment, with a degree of reversibility demonstrated for those receiving IMT [121]; whilst visual field defects appear to progress over time among patients with active disease, some improvement has been observed in patients in remission [63, 64].

Recently, Arya and collegues applied pointwise linear regression (PLR) analysis to results of automated HVF in patients with BCR, and were able to identify field loss in patients despite a stable MD, and stable and even normal electrophysiology results [122]. This is in line with the results of Tomkins-Netzer et al. which suggest that Pattern Standard Deviation (PSD) is more sensitive than MD in detecting deterioration in BCR, and advised that such objective metrics of permetric function should be a standard adjunct to electrophysiology in characterisation of retinal dysfunction in BCR [74].

## Patient partnership

Like a number of other rare diseases, research in BCR has benefitted from the support and engagement of dynamic patient groups with national and, increasingly, international reach. International conferences on BCR often have strong patient involvement (http://www.brcophthalmology.org/events/birdshot-patient-day-2010, http://www.uveitis.org/news/post/2nd-international-symposium-on-birdshot-retinochoroidopathy). Indeed the UK ‘Birdshot Days’ are entirely organized by patients but with an invited scientific panel of experts. Koutroumanos et al. undertook formal feedback from attendees at the inaugural UK ‘Birdshot Day’ (50 patients, 26 carers and a multidisciplinary group of 50 health professionals) in 2010. They found that patients, carers and professionals all felt significantly educated by the event, that the sense of isolation felt by patients was reduced and that networking was developed among all attendees. Such events are also critical in providing an opportunity for patients to communicate their priorities for future research, and influence the research agenda [123]. Koutroumanos noted that in response to the question ‘Assuming there is no cure, what is the single factor that would improve the quality of my life the most?’, the leading priorities were ‘Fewer drug side-effects’ (56 %), ‘More frequent and detailed monitoring’ (23 %), ‘Practical or financial support’ (9 %) or ‘Emotional support’ (5 %). When patients and their carers were asked what they wished current research would concentrate on, the leading priorities were ‘Finding out what causes the disease’ (48 %), ‘Better medicines’ (32 %), *‘*Faster and more accurate diagnosis’ (13 %) and ‘Better monitoring’ (7 %) [123]. One of the outcomes from this patient-professional partnership has been the development and validation of novel patient reported outcome measures for BCR, with separate questionnaires to capture key symptoms, quality of life and impact of medication [124]. The authors note the potential value of such tools to ensure (1) a more holistic approach to patient care, and (2) that future clinical studies in BCR assess patient-relevant outcomes that capture the range of patient experience-not only any improvement in symptoms and visual function but also any potential negative impacts arising from the intervention.

## Conclusion

Recent advances in our understanding of the pathophysiology of BCR have identified several interesting signaling pathways on which to target future therapeutic agents. Furthermore, the detection of peripheral cytokines involved in these pathways may enable their use as biomarkers for disease progression, response to therapy, and disease stratification.

Traditional protocols for monitoring of BCR with ERG and perimetry are now being supplemented by multi-modal imaging. Detailed prospective evaluation is required to assess the relationship between the structural abnormalities of the retina and choroid revealed by enhanced depth and extramacular OCT, OCTA, FAF, FA and ICG, and the downstream impact on visual function. Such tools will provide the more sensitive outcome measures we need to facilitate high quality interventional trials in BCR, and to provide the evidence that will inform ‘best practice’ for monitoring and treatment of patients with BCR.

## Abbreviations

BCR: birdshot chorioretinopathy
CMO: cystoid macular oedema
CsA: cyclosporine A
ERG: electroretinography
FA: fluorescein angiography
FAF: Fundus auto fluorescence
HLA-B29: human leucocyte antigen-b29
ICG: Indocyanine green angiography
IL: interleukin
IMT: Immunomodulatory therapy
MMF: Mycophenolate mofetil
OCT: optical coherence tomography
S-Ag: retinal S-antigen
TGF-β1: transforming growth factor-β1
Th: T-helper
TNF-a: tumor necrosis factor-a
